# A novel technique for central hepatectomy: Maintain the blood supply and biliary drainage on one side and the blood supply from the portal vein on the other

**DOI:** 10.3892/etm.2013.1367

**Published:** 2013-10-29

**Authors:** CHUN TANG, JUN-TAO YANG, HONG-XU CHEN, XIAN-CHUN LIANG, HONG-MING LIU, PING CHEN

**Affiliations:** Department of Hepatobiliary Surgery, Daping Hospital, Third Military Medical University, Institute of Surgery, Chongqing 400042, P.R. China

**Keywords:** middle lobe liver tumor, middle hepatectomy, biliary drainage, blood supply

## Abstract

Central hepatectomy is amongst the most difficult surgeries of liver tumors. For the routine local excision of a tumor, if the tumor has invaded the blood vessels or bile duct of the liver, then half of the liver or three lobes of the liver are resected. This results in two major drawbacks, one of which is that the residual hepatic lobe may not compensate for the damage, so it is not possible to perform conventional partial resection. The other is that the volume of normal liver tissue removed may be much more than the volume of tumor removed, causing substantial waste. In the present study, surgery was performed to resect a central liver tumor. In that surgery, the V segment and parts of the IV, VI and VIII segments were resected, and the blood supply and biliary drainage of the left hepatic lobe were kept intact. However, for the remaining VI, VII and VIII segments of the right hepatic lobe, only the blood supply from the portal vein was maintained and no arterial blood supply or biliary drainage was kept so that the patient had the opportunity to undergo radical resection and successful rehabilitation. The reason these opportunities may be possible is that the residual right liver is a temporary replacement therapy in the perioperative period. Therefore, for central hepatic tumors, particularly tumors that have invaded the neighboring bile ducts or blood vessels, if the blood supply and biliary drainage on one side is maintained and the blood supply to the other side from the portal vein is kept intact, then it is possible to perform radical resection. This provides a novel approach to the clinical resection of central liver tumors.

## Introduction

The site of liver tumors is no longer an issue with resection due to the advances of surgical techniques and instruments. The decisive factor in the radical resection of liver tumors is whether the remnant liver is able to meet the needs of the body. For left and right lobe liver tumors it is easy to assess the resection volume and residual liver volume, but the surgical treatment of central liver tumors is more difficult. Radical resection is required for the treatment of hilar cholangiocarcinoma and for tumors which have invaded liver blood vessels or bile ducts, and usually half of the liver or three liver lobes are resected. This type of surgery results in two major drawbacks.

One drawback is that if the residual liver does not meet the physical needs of the body, then it is not possible to perform radical resection. Also, certain specialized treatments for liver function replacement are required if radical resection is performed and currently there are three which are commonly used: orthotopic liver transplantation, auxiliary liver transplantation and bioartificial liver replacement therapy.

For cancer patients who are able to undergo liver transplantation, orthotopic liver transplantation is the best choice (1,2). However, with the current shortage of donor livers, the lack of donor sources is the most significant limitation of this treatment (3) and therefore orthotopic liver transplantation only meets the requirements of a few people. In addition, the high cost of the long-term use of anti-rejection drugs also limits the wide application of the treatment in clinical practice.

Auxiliary liver transplantation, regardless of whether it is homologous or heterologous or whether the whole liver or part of it is transplanted, may result in immune rejection (4). Immune rejection is particularly prominent in heterologous auxiliary liver transplantation (5). Further, there is the issue of a donor source in homologous auxiliary liver transplantation. As the original liver function is gradually restored, a number of the donor livers will require surgical resection, which will increase the trauma of the patient. All of the aforementioned issues limit the wide application of this treatment in clinical practice.

A bioartificial liver support system consists of two core components: cells and a bioreactor. The whole blood or plasma of a patient interacts directly or indirectly with the cells in the bioreactor, which have characteristics of primary liver cells, and liver functions, such as liver detoxification, synthesis, biological transformation and other support functions, are achievable (6,7). Human liver cells are the most ideal cells for use in a bioartificial liver support system, but human liver cell shortage is the biggest obstacle of this type of therapy. Most bioartificial liver support systems use pig liver cells, which has resulted in a number of major issues, including immune rejection and zoonotic diseases (8). Bleeding, infection and other complications have also been observed in patients treated with these systems (9,10). Furthermore, the cost of a bioartificial liver support system is quite high. All of these issues limit the wide-spread application of bioartificial liver support systems in clinical practice.

The other major drawback regarding the treatment of central liver tumors is that half of the liver or three liver lobes, which is often far more than the total tumor volume, is resected. This type of resection causes a large amount of waste, which is particularly evident in the treatment of hilar cholangiocarcinoma. Tumors in central liver cancer are mainly located in the middle of the IV, V and VIII segments, while the majority of the surrounding liver tissue is normal. The reason that half of the liver or three liver lobes are resected is mainly due to tumor invasion to the first hepatic portal vein and portal venous system. Complete resection of a tumor in the liver easily leads to a deficient blood supply or biliary drainage problem, so the surrounding normal liver tissue has to be resected. Based on that, a study has suggested that only a small amount of the surrounding normal liver tissue be resected in the case of curative resection (11). Although this ensures that most of the normal liver tissue is retained and effectively reduces complications, this method has two major shortcomings: the bile duct stump is difficult to handle and it is difficult to ensure the arterial blood supply. Therefore, a skilled surgeon is required.

Consequently, a novel and simple surgical procedure is required. In the present study, a unique method of central liver tumor resection was performed. The V segment and parts of the IV, VI and VIII segments were resected, and the blood supply and biliary drainage of left hepatic lobe were maintained, but for the remaining VI, VII and VIII segments of the right hepatic lobe, only the blood supply from the portal vein was kept intact and no arterial blood supply or biliary drainage was kept. This method ensured that the patient had the opportunity to undergo radical resection and successful rehabilitation, and it also provided a novel approach to the clinical resection of central liver tumors.

## Case Report

**Figure 1:**
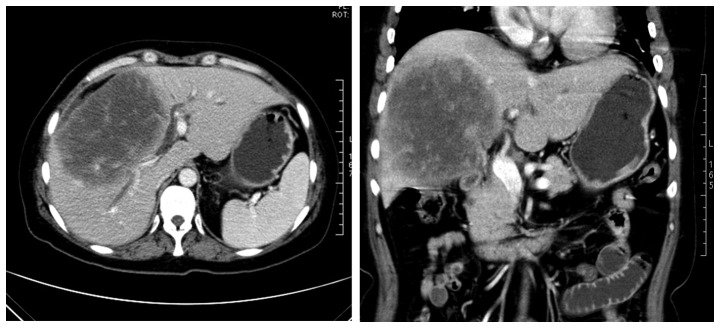
CT images of a patient with large liver tumor. The right hepatic bile duct was invaded and the left bile duct was slightly expanded due to tumor compression.

A 49-year-old female who had experienced recurrent upper abdominal pain for eight months was admitted to Department of Hepatobiliary Surgery, Daping Hospital, Third Military Medical University, Institute of Surgery (Chongqing, China) on May 10, 2011 following an increase in pain with jaundice for >10 days. The blood test results of the patient were as follows: total bilirubin (TBIL), 297.0 μmol/l; direct bilirubin (DBIL), 159.2 μmol/l; glutamic-oxalacetic transaminase (AST), 131.1 U/l; glutamic-pyruvic transaminase (ALT), 101.5 U/l; and albumin (ALB), 32.4 g/l. The serum tumor marker levels of the patient were as follows: CA125, 82.24 U/l; CA153, 28.6 U/l; and CA199, 144.06 U/l. The hepatitis marker test results were negative. CT examination revealed a large tumor in the liver that had invaded the right hepatic bile duct, and the left bile duct was slightly expanded due to the tumor invasion (Fig. 1). According to conventional methods of treatment, radical resection is required and the right liver or the right three lobes should be resected, in which case the residual liver may not be able to meet the needs of the body and may harm postoperative recovery. Therefore, in the present study, the central liver was resected with the right hepatic bile duct and a biliary-enteric anastomosis was performed on the right hepatic ducts.

**Figure 2:**
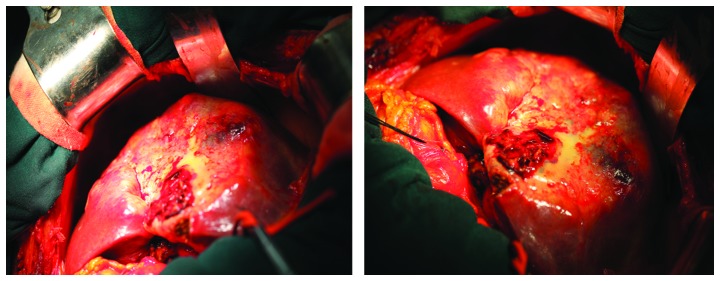
Intraoperative tumor probing. Intraoperative tumor probing. A large tumor was observed in the central liver with hard texture and approximate 15 cm diameter, which invaded the whole V segment and parts of the IV, VI and VIII segments. The hilar lymph nodes were enlarged with a slightly hard texture.

The surgery was performed on May 12, 2011. A large tumor was observed in the middle liver lobe (see Fig. 2) and the hilar lymph nodes were enlarged with a slightly harder texture than normal.

Following opening of the common bile duct, a 5-mm cholangioscope was used to explore the bile duct and it detected that the distal common bile duct was unobstructed, the left hepatic duct was slightly narrowed (possibly as a result of compression by the tumor) but the cholangioscope was able to pass through, the mucosa was normal, and the right hepatic duct was also narrowed and the cholangioscope was not able to pass through. The hepatic portal system was carefully dissected and it was observed that the left hepatic bile duct, artery and portal vein were completely dissected; the right hepatic portal vein could be dissected, but the right hepatic artery and bile duct could not be dissected due to tumor invasion. Therefore, the central liver was resected with the right hepatic bile duct and artery, the hilar lymph nodes were removed and a biliary-enteric anastomosis was performed on the right hepatic ducts.

**Figure 3:**
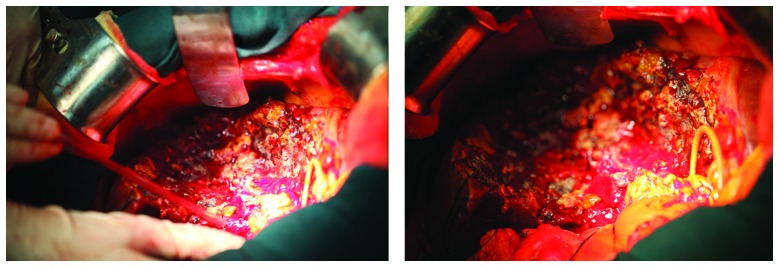
Following tumor resection. Following tumor resection. The tumor was completely resected 2 mm around the edge of the tumor using cutting forceps. Resection range included the V segment and parts of the IV, VI and VIII segments. The left hepatic artery, portal vein, bile duct and right hepatic portal vein were kept intact. A bile duct stump of ~0.2 mm diameter was identified in the residual VII segment.

Following removal of the hilar lymph nodes, the right hepatic artery and bile ducts were ligated and cut off, the hepatic portal system was completely blocked and the tumor was completely resected 2 mm around the edge of the tumor using cutting forceps (Fig. 3).

Following careful examination of the left and right hepatic sections, a bile duct stump of ~0.2 mm diameter was identified in the residual VII segment. This duct stump was too thin for the biliary-enteric anastomosis to be performed on, as it would easily lead to complications, such as bile leakage, bile duct stricture and cholangitis. Therefore, it was ligated without performing biliary-enteric anastomosis. Following careful hemostasis, single lumen drainage was performed, T-tube drainage was used for the common bile duct and the abdomen was closed.

On the first day following surgery, the vital signs of the patient were stable, the T-tube drained ~50 ml of yellow fluid and ~150 ml of bloody fluid was drained from the liver sections. The blood test results of the patient were as follows: TBIL, 203.1 μmol/l; DBIL, 123.6 μmol/l; AST, 526.4 U/l; ALT, 347.6 U/l; and ALB 28.2 g/l.

**Figure 4:**
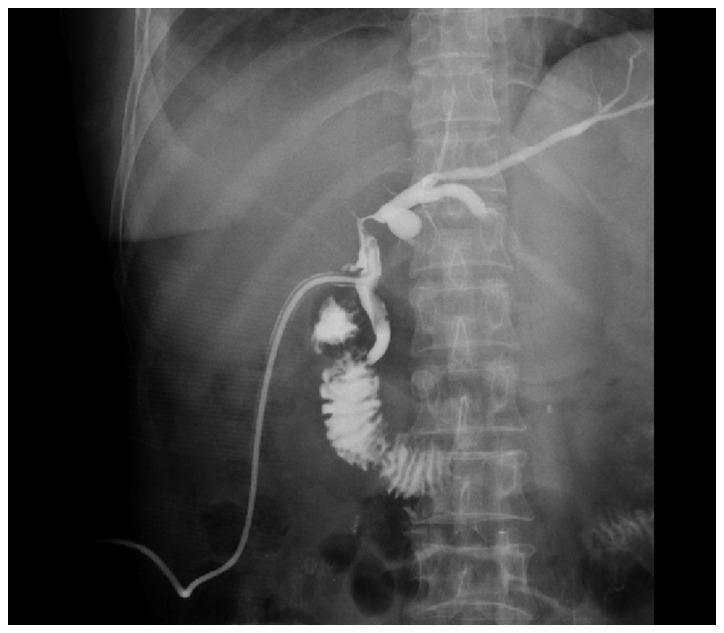
Following surgery (40 days), imaging of the T tube revealed a clear common bile duct, the left bile duct was visible and the right hepatic bile duct was missing.

In the following days, the T-tube drainage gradually increased and the color of the fluid became darker, while drainage from the liver sections gradually decreased. On fifth day following surgery, no fluid was drained from the liver sections and ~200 ml of liquid was drained by the T-tube. The blood test results of the patient were as follows: TBIL, 133.0 μmol/l; DBIL, 75.9 μmol/l; AST, 27.5 U/l; ALT, 60.0 U/l; and ALB 30.7 g/l. Drainage of the liver sections was removed six days after surgery and the patient was discharged from hospital with the T-tube drainage. The T-tube drainage was occluded 30 days after surgery and the patient experienced no discomfort. At 10 days after the T-tube occlusion, a cholangiogram image of the T-tube revealed that the distal common bile duct was unobstructed and the left hepatic duct was clearly visible on the image, but the right hepatic duct was completely absent from the image (Fig. 4). Subsequently, the T-tube was removed and the patient lived well for five months following the surgery, after which time contact with the patient was lost. If the patient passed away at that time, the survival time was longer than it was likely to have been if the surgery had not been performed.

## Discussion

The residual liver of the patient treated in the present study had two features: the full blood supply and left hepatic lobe with biliary drainage were kept, and for the remaining VI, VII and VIII segments of the right hepatic lobe, only the blood supply from the portal vein was maintained and no arterial blood supply or biliary drainage was kept. There are two main reasons why the patient rehabilitated successfully.

One reason is that the residual left hepatic lobe was able to compensate for the damage and meet the needs of the body. Due to the limited conditions of the hospital in which the patient was treated, it was not possible to accurately assess the volume of the residual left hepatic lobe, but by analyzing the patient’s preoperative CT scan it was considered that the residual left hepatic lobe would not be able to meet the needs of the body. Therefore, it is likely that the residual right hepatic lobe with blood supply from portal vein contributed to the patient’s successful rehabilitation. In the present case, it was not possible to perform a biliary-enteric anastomosis as the bile duct stump of the right hepatic lobe was tiny. In most cases, if it is not possible to perform a biliary-enteric anastomosis then the residual right hepatic lobe is resected. However, this may result in acute liver failure, which may affect the patient’s rehabilitation. The major functions of a normal liver include metabolism, bile formation and excretion, detoxification, immunity, blood coagulation, regulation of blood volume in the body, generation of heat, and regulation of water and the electrolyte balance. In the present study, the blood supply and biliary drainage of left hepatic lobe were kept intact, but for the remaining right hepatic lobe, only the blood supply from the portal vein was maintained. Seventy percent of the liver’s blood supply comes from the portal vein system, so maintaining this blood supply in the residual right hepatic lobe prevented acute hepatic necrosis (8,12,13) and maintained the vast majority of the functions of this lobe, with the exception of bile excretion. The whole blood supply of the intrahepatic bile ducts is from the concomitant hepatic artery (14), and the left and right hepatic arteries have no anastomotic branches; thus, the bile duct cells are the most vulnerable to hypoxic-ischemic necrosis due to the hepatic artery injury. Once the hepatic artery is cut off, collateral circulation forms in the body through the right inferior phrenic artery, left gastric artery, inferior pancreaticoduodenal artery, superior mesenteric artery, gastroduodenal artery and so on (15). Tumor invasion of the hepatic artery is a chronic process in which the hepatic artery becomes gradually narrower due to the tumor’s involvement and its collateral circulation gradually develops. Therefore, an artery of the liver affected by tumor invasion is not likely to affect the blood supply of the bile duct cells in the majority of patients. Conjugated bilirubin synthesized by the residual right liver is able to move to the left of the liver with the blood circulation and be discharged to the biliary system. Therefore the residual right liver was involved in temporary replacement therapy in the perioperative period, which ensured that no acute liver failure occurred. Over time, the residual right liver gradually shrank due to cholestasis and biliary cirrhosis, but compensatory hyperplasia occurred in the patient’s left hepatic lobe, which ensured that the patient eventually recovered.

Based on this analysis, the following hypothesis was formed. In the hepatic vascular system, the portal vein is thick and its distal-end diameter is able to meet the needs of normal vascular anastomosis. Therefore, it is crucial to keep the distal hepatic portal vein in surgery. For central hepatic tumors, particularly tumors that have invaded the neighboring bile ducts or blood vessels, when the residual liver would not be able to meet the needs of the body if half-liver or three-liver lobe resection was performed, radical resection of the tumor and one-side bile duct or blood vessel should be performed. It would be favorable for biliary-enteric anastomosis or anastomosis to be applied, but if the residual bile duct or artery is very small, anastomosis bypass may be applied in the thicker portal vein system so that the residual liver is a temporary replacement therapy and is able to help the patient avoid acute liver failure in the perioperative period. This hypothesis needs to be validated and, therefore, in a future study, strict postoperative follow-up will be applied and animal experiments will be performed.

The animals will be randomly divided into two groups: lethal right liver resection without blood supply from the hepatic portal vein will be performed in one group and lethal right liver resection with blood supply from the hepatic portal vein will be performed in the other group. The results will be analyzed and the hypothesis evaluated. If the hypothesis is true, a novel method for the clinical resection of central liver tumors will be achieved and more patients with central liver tumors are likely to benefit from improved treatment.
